# Integrated single-cell and bulk RNA dequencing to identify and validate prognostic genes related to T Cell senescence in acute myeloid leukemia

**DOI:** 10.3389/fbinf.2025.1606284

**Published:** 2025-06-25

**Authors:** Mengyao Sha, Jun Chen, Haifeng Hou, Huaihui Dou, Yan Zhang

**Affiliations:** ^1^ Department of Laboratory Medicine, Suzhou Yongding Hospital, Suzhou, China; ^2^ Department of Hematology, Suzhou Yongding Hospital, Suzhou, China

**Keywords:** acute myeloid leukemia, T cell, cell senescence, single-cell RNA sequencing, prognostic risk model

## Abstract

**Background:**

T-cell suppression in patients with Acute myeloid leukemia (AML) limits tumor cell clearance. This study aimed to explore the role of T-cell senescence-related genes in AML progression using single-cell RNA sequencing (scRNA-seq), bulk RNA sequencing (RNA-seq), and survival data of patients with AML in the TCGA database.

**Methods:**

The Uniform Manifold Approximation and Projection (UMAP) algorithm was used to identify different cell clusters in the GSE116256, and differentially expressed genes (DEGs) in T-cells were identified using the FindAllMarkers analysis. GSE114868 was used to identify DEGs in AML and control samples. Both were crossed with the CellAge database to identify aging-related genes. Univariate and multivariate regression analyses were performed to screen prognostic genes using the AML Cohort in The Cancer Genome Atlas (TCGA) Database (TCGA-LAML), and risk models were constructed to identify high-risk and low-risk patients. Line graphs showing the survival of patients with AML were created based on the independent prognostic factors, and Receiver Operating Characteristic Curve (ROC) curves were used to calculate the predictive accuracy of the line graph. GSE71014 was used to validate the prognostic ability of the risk score model. Tumor immune infiltration analysis was used to compare differences in tumor immune microenvironments between high- and low-risk AML groups. Finally, the expression levels of prognostic genes were verified using polymerase chain reaction (RT-qPCR).

**Results:**

31 AMLDEGs associated with aging identified 4 prognostic genes (CALR, CDK6, HOXA9, and PARP1) by univariate, multivariate, and stepwise regression analyses with risk modeling The ROC curves suggested that the line graph based on the independent prognostic factors accurately predicted the 1-, 3-, and 5-year survival of patients with AML. Tumor immune infiltration analyses suggested significant differences in the tumor immune microenvironment between low- and high-risk groups. Prognostic genes showed strong binding activity to target drugs (IGF1R and ABT737). RT-qPCR verified that prognostic gene expression was consistent with the data prediction results.

**Conclusion:**

CALR, CDK6, HOXA9, and PARP1 predicted disease progression and prognosis in patients with AML. Based on these, we developed and validated a new AML risk model with great potential for predicting patients’ prognosis and survival.

## 1 Introduction

Acute myeloid leukemia (AML) is a heterogeneous hematological neoplasm characterized by the clonal proliferation of myeloid cells in peripheral blood and bone marrow (Pollyea et al., 2021), with a 5-year survival rate of less than 30% (Narayanan and Weinberg, 2020). Cellular senescence is an irreversible state of cell cycle arrest characterized by DNA damage, telomere shortening, and secretion of pro-inflammatory cytokines (Rossiello et al., 2022). Recent studies have emphasized that T-cell dysfunction, including senescence, is a key contributing factor to immune evasion and treatment resistance in AML (Chen et al., 2025; Ikeda et al., 2025; Yuan et al., 2021), as evidenced by the poor prognosis and low survival of patients with AML (Mazziotta et al., 2024).

Previous studies have identified markers to study T-cell senescence in some patients with AML, such as increased KLRG1 expression (Shive et al., 2021; Towers et al., 2024). These studies focused on identifying senescence markers; however, knowledge of the impact of T-cell senescence and subpopulation changes on the immune microenvironment of AML tumors and AML prognosis remains inadequate. In this study, we identified AML prognostic genes related to T-cell senescence using transcriptome (GSE116256) and single-cell sequencing (GSE114868) data and constructed a prognostic risk model. We also developed a predictive line graph for viability using TCGA-LAML and another transcriptome data (GSE71014). We then analyzed the differences in the immune microenvironment between the low- and high-risk AML groups. The relationship between T cell subsets and prognostic genes, and which T cell subsets were most significantly associated with AML, was evaluated, predicting target lncRNAs, miRNAs, and drugs. Finally, we concluded that prognostic gene expression influences T cell senescence, altering the immune microenvironment. This causes tumor immune escape, thereby determining the prognosis and survival duration of patients with AML. Our findings provide new insights into the role of T-cell senescence in AML and suggest potential therapeutic targets for improving immunotherapy-based treatments.

## 2 Materials and methods

### 2.1 Data collection

We downloaded gene expression data from the GSE114868, GSE116256, and GSE71014 datasets associated with AML through the Gene Expression Omnibus (GEO) repository (http://www.ncbi.nlm.nih.gov/geo/). Eight hundred and sixty-six cellular senescence-related genes (CSRGs) were extracted from the CellAge database (Avelar et al., 2020; Chatsirisupachai et al., 2019) (https://genomics.senescence.info/cells/) (Supplementary Table S1). The survival data and gene expression profiles of the TCGA-LAML cohort were obtained from the TCGA database (https://xena.ucsc.edu/) (Table 1).

**TABLE 1:** Dataset collection.

Data source	Experimental cohort	Control cohort	Platform
GSE114868	194 bone marrow mononuclear cell samples from AML patients	20 normal control human bone marrow mononuclear cell samples	GPL17586
GSE116256	16 Bone marrow samples from AML patients	5 bone marrow samples from normal controls	GPL18573
TCGA-LAML	132 blood samples from AML patients with survival information		——
GSE71014	104 bone marrow samples from AML patients		GPL10558
Senescence gene set	866 CSRGs were obtained from the CellAge database		——

### 2.2 Differential expression analysis

Differentially expressed genes (DEGs) in the AML and control groups from the GSE114868 dataset were identified using the Limma software package (v 3.54.0). All hypothesis tests were corrected using the Benjamini–Hochberg method to control the false discovery rate (FDR <0.05), and the screening criteria for differentially expressed genes were |log 2 fold change (FC)|>1, adj. *p* < 0.05 (Ritchie et al., 2015). The ggplot2 (Gustavsson et al., 2022) and ComplexHeatmap (v 2.14.0) (Gu and Hübschmann, 2022) packages were used to visualize the top ten significantly DEGs via a volcano plot and a heatmap, respectively.

### 2.3 Single-cell analysis

In the GSE116256 scRNA-seq dataset, cells were curated using Seurat’s NormalizeData function (Gu and Hübschmann, 2022), selecting high-quality cells based on stringent criteria (nFeature RNA >200, genes expressed in <3 cells removed, nCount RNA between 200–3,000, and mitochondrial proportion <10%). The FindVariableFeatures function identified the top 2,000 highly variable genes (HVGs). Subsequent normalization and PCA outlier detection were conducted using Seurat’s Scale Data function, with Elbowplot visualization and Jackstraw reclustering used to determine the top 30 significant principal components (PCs) (*p* < 0.05). Dimensionality reduction and clustering were performed using a UMAP with 30 PCs and Seurat’s FindNeighbors and FindClusters functions (resolution = 0.4). Notable marker genes for distinct clusters were identified using the FindAllMarkers function (logfc. Threshold = 0.5, min. pct = 0.25, return. thresh = 0.01). Cell annotation was assigned based on these markers using SingleR (v 2.4.0) (Aran et al., 2019) and the CellMarker database. Finally, to investigate the differences in gene expression at the T cell level between AML and controls, we screened the DEGs of T cells in AML and controls using the FindMarkers function. We depicted them using Manhattan plots (|log2 FC| > 0.1, adj. *p* < 0.05).

### 2.4 Identification and functional analysis of candidate genes and protein-protein interaction (PPI) networks

We used the VennDiagram package (v 1.7.3) (Chen and Boutros, 2011) to intersect T cell DEGs with CSRGs and GSE114868 DEGs. The ClusterProfiler package (v 4.7.1.3) (Yu et al., 2012) was used to perform Gene Ontology (GO) and Kyoto Encyclopedia of the Genome (KEGG) enrichment analyses (*p* < 0.05). We visualized the data using the GO plot (v 1.0.2) (Walter et al., 2015) and enrichment (v 1.22.0) packages (Wang et al., 2022). The interacting gene search tool (STRING database [http://www.string-db.org/]) and Cytoscape software (v 3.7.2) (Liu et al., 2020) were used to build protein-protein interaction (PPI) networks with a confidence threshold >0.4.

### 2.5 The screening of the prognostic genes and construction of a risk model

We refined candidate genes linked to survival and prognosis using a univariate Cox regression analysis (HR ≠ 1, *p* < 0.05). We validated the proportional hazards (PH) assumption (*p* > 0.05) using the survival package (v 3.7-0) (Lei et al., 2023) and cox. zph function on TCGA-LAML data. This was followed by multivariate and stepwise Cox regression analyses (*p* < 0.05) to identify prognostic genes for AML risk model development.

$$risk\;score=\sum_{i=1}^{n}coef\left({gene}_{i}\right)\times expr\;\left({gene}_{i}\right)$$

Where coef is the gene regression coefficient and expr is the gene expression level.

Patients in the TCGA-LAML and GSE71014 databases were categorized into high- and low-risk groups based on the median risk score. We plotted risk and Kaplan-Meier Survival Curves using the survminer package (v 0.4.9) (Ramsay et al., 2018). The AUC was determined by ROC analysis of the 1-, 3-, and 5-year survival using the survivalROC package (v 1.0.3.1) (Heagerty et al., 2000).

### 2.6 Independent prognostic analysis and line graph

We performed a univariate Cox regression analysis, which included risk scores, age, and type. A HR≠1 and *p* < 0.05 represented the prognostic significance of the model, and the model met the proportional hazards assumption (*p* > 0.05). We used the survival package (v 3.7-0) for the multivariate analysis and the rms package (v 6.5.0) (Heagerty et al., 2000) to construct line plots to predict the 1-, 3-, and 5-year survival of patients with AML. We plotted ROC curves to verify the reliability of the line graphs using the plotROC package (v 2.3.1) (Sachs, 2017).

### 2.7 Tumor immune microenvironment analysis

The estimate package (v 1.0.13) and Wilcoxon test assessed the AML immune microenvironment. The ssGSEA (Charoentong et al., 2017) and GSVA (v 1.46.0) packages (Hänzelmann et al., 2013) calculated the enrichment of 28 immune cell populations, and the Wilcoxon test compared their ratios in TCGA-LAML samples (*p* < 0.05). We performed a Spearman correlation analysis using the psych package (v 2.4.3) to assess the association between immune cells and prognostic genes (|cor| > 0.30, *p* < 0.05).

### 2.8 Regulatory network construction

We used the MultiMiR package (v 1.24.3) (Ru et al., 2014) to predict mRNAs targeted by miRNAs based on the TargetScan and PITA databases. We used starBase (https://rnasysu.com/encori/) to identify the upstream lncRNAs of miRNAs and visualized “lncRNA-miRNA-mRNA” networks using Cytoscape (v 3.9.1) (Liu et al., 2020). We explored prognostic gene interactions and co-expression using the geneMANIA (https://genemania.org/) database.

### 2.9 Chromosomal and subcellular localization

The chromosomal location of prognostic genes was determined using ENSEMBL (https://asia.ensembl.org/index.html) and mapped to chromosomes using the RCircos package (v 1.2.2) (Zhang et al., 2013). Proteins were extracted from Genecards (https://www.genecards.org/) sequences to study the subcellular localization of proteins encoded by prognostic genes, and the ggplot2 package (v 3.4.1) was used to visualize the cellular distribution of the proteins.

### 2.10 Drug prediction and molecular docking

We assessed drug sensitivity using the GDSC database. The pRRophetic package (v 0.5) (Geeleher et al., 2014) determined the IC50 values, and a Wilcoxon test compared drug sensitivities between different risk groups (*p* < 0.05). The corrplot package (v 0.92) (Wang et al., 2022) performed a Spearman’s test (|cor| > 0.30, *p* < 0.05) to analyze the correlation between IC50 values and gene expression. Highly correlated 3D drug structures were obtained from the PubChem and RSCB PDB databases. CB-Dock2 was used to evaluate affinity, with a binding energy below −5 kcal/mol indicating affinity.

### 2.11 Experimental validation by RT-qPCR

Bone marrow samples from controls and patients with AML were collected at Suzhou Yongding Hospital, and informed consent and ethical approval were obtained (202450; see Supplementary Table S3 for sample details).

### 2.12 Statistical analysis

Data processing and comparisons were performed using R (v 4.2.3), with statistical significance determined by Wilcoxon tests at *p* < 0.05.

## 3 Results

### 3.1 Five hundred and seventeen T-cell DEGs identified in the scRNA-seq dataset GSE116256 and T-cell subcluster analysis

**FIGURE 1:**
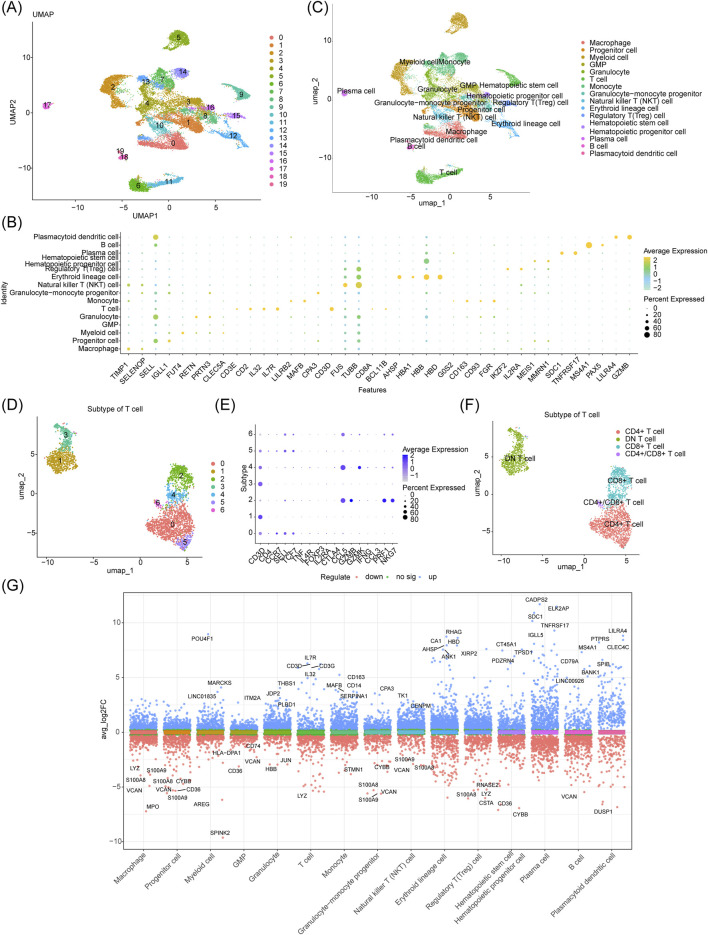
Five hundred and seventeen T-cell DEGs identified in the scRNA-seq dataset GSE116256 and T-cell subcluster analysis. **(A)** UMAP plot of 20 different cell clusters obtained by dimensionality reduction clustering before annotation. **(B)** Bubble plot of gene expression of different cellular markers. **(C)** The 15 major cell types identified after annotation. **(D–F)** Further dimensionality reduction and clustering of T cell cluster 6 revealed four distinct T cell types. **(G)** Manhattan plot of 517 DEGs in T cell clusters.

The GSE116256 dataset contained 21,253 cells and 19,003 genes for subgroup identification and annotation afterquality control (QC) (Supplementary Figure S1A). Supplementary Figure S1B shows the inclusion of 2,000 high-variance genes (red dots). Supplementary Figure S1C shows no outlier samples in GSE116256. An elbow plot was used to visualize the 2,000 high-variance genes after principal component analysis. The optimal number of dimensions for cell clustering was determined to be 30 (*p* < 0.05) using linear dimensionality reduction (Supplementary Figure S1D,E). After co-clustering the cells into 20 different cell clusters (Figure 1A), bubble plots showed the expression of typical marker genes in 15 cell clusters (Figure 1B, Supplementary Table S2). The annotated cell groups are shown in Figure 1C. Among them, monocytes had three clusters (7, 13, and 14), and T cells had two clusters (6 and 11). After a differential analysis of the genes in the subclustered cells, clusters 7 and 13 were shown to contain 340 and 736 DEGs (cluster 14 did not), respectively, based on the screening according to predefined criteria (|logFC| > 0.1 and adj. *p* < 0.05; Supplementary Figure S2A,B). A functional analysis revealed that the two clusters of monocytes were involved in significantly different biological processes and pathways (Supplementary Figure S2C–F). Given that the impact of T-cell senescence and subpopulation changes in the AML tumor immune microenvironment is still poorly understood, we clustered the T-cell subpopulations based on T-cell-typical marker gene expression (Szabo et al., 2019) (Figures 1D,E). The annotated cell clusters are shown in Figure 1F. The Manhattan plot suggests 517 T-cells DEGs between the control and AML groups (358 upregulated and 159 downregulated genes) (Figure 1G; Supplementary Table S4).

### 3.2 Screening of AML candidate genes based on the GSE114868, GSE116256, and CellAge databases

**FIGURE 2:**
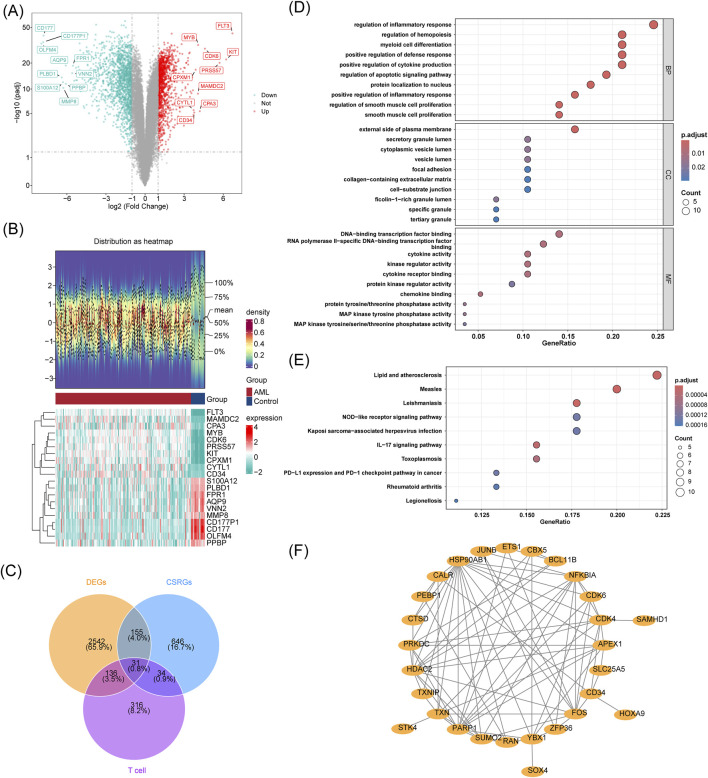
Screening of AML candidate genes based on the GSE114868, GSE116256, and CellAge databases. **(A)** Volcano plot of DEGs in GSE114868, with genes labeled according to significance; **(B)**The heatmap consists of two parts: the upper part shows the density plot of DEGs expression with lines representing five quantiles and the mean; the lower part is the DEGs expression heatmap (red for AML group, green for Control group). **(C)** Venn diagram showing the intersection of DEGs from GSE116256, GSE114868, and CSRGs; **(D–F)** GO/KEGG enrichment and PPI network analysis of 31 cross-genes.

The GSE114868 dataset yielded 2,864 DEGs (downregulated: 1,552; upregulated: 1,312), with the top ten DEGs highlighted in the volcano and heat maps (Figures 2A,B). The intersection between the 2,864 DEGs with 866 CSRGs and 517 T-cell DEGs yielded 31 candidate genes (Figure 2C; Supplementary Table S5). The enrichment analysis and PPI networks of the 31 candidate genes is shown in Figures 2D–F.

### 3.3 Establishment and evaluation of a prognostic model for AML based on the TCGA-LAML cohort and validation of a prognostic model for AML based on GSE71014

**FIGURE 3:**
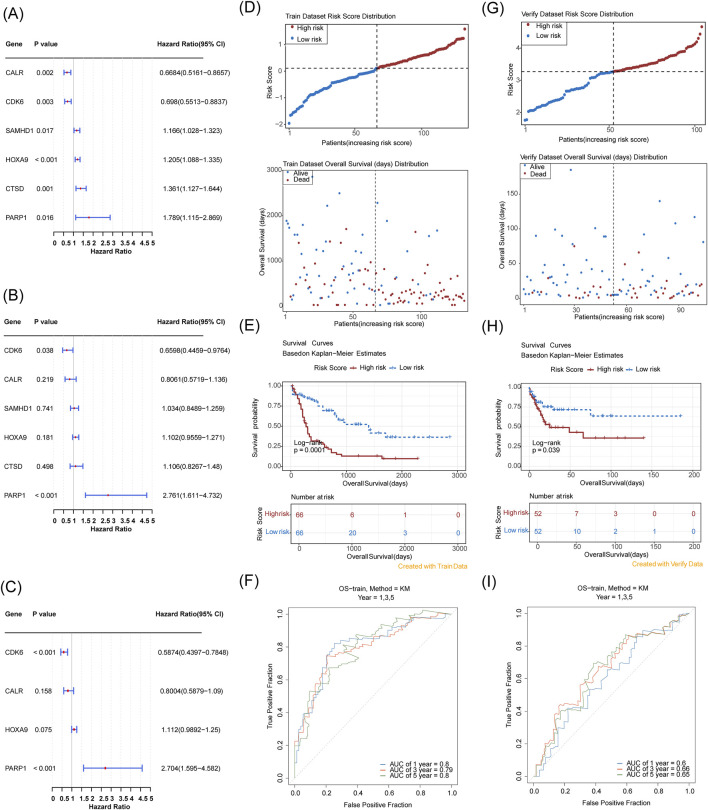
Establishment and evaluation of a prognostic model for AML based on the TCGA-LAML cohort and validation of a prognostic model for AML based on GSE71014. **(A)** Six prognostic genes identified via univariate analysis; **(B,C)** Four prognostic genes determined by multivariate Cox and stepwise regression analyses; **(D)** Risk score distribution and Survival status distribution (The x-axis of both panels represents patients sorted by risk score. The y-axis of the upper panel shows risk scores, with a dashed line indicating the median risk score and its corresponding patient count. The y-axis of the lower panel shows survival time, with a dashed line indicating the median risk score and its corresponding patient count). **(E)** Kaplan-Meier curve; **(F)** 1-, 3-, 5-year ROC curves; **(G–I)** Validation in GSE71014 with corresponding risk score and survival analysis, and ROC curve assessment at 1, 3, and 5 years.

Using multifactorial models that directly incorporate too many variables resulted in model instability and overfitting. Therefore, we first performed a univariate Cox regression analysis on the TCGA-LAML cohort (HR = 1: no significant effect; HR < 1: protective genes; HR > 1: risk genes; *p* < 0.05) to initially screen for variables that were significantly associated with AML survival. The results of the univariate Cox regression analysis were then subjected to the proportional hazards (PH) hypothesis test (*p* > 0.05) (Supplementary Figure S3), and six genes were finally identified: CALR, CDK6, CTSD, HOXA9, PARP1, and SAMHD1 (Figure 3A). A multifactor Cox model was constructed for the six genes to obtain a more robust prediction model (Figure 3B). A stepwise regression analysis was performed based on the multifactor Cox regression analysis (Figure 3C), which resulted in the final identification of four prognostic genes: CALR, CDK6, HOXA9, and PARP1. The formula for the risk model was

$$\begin{array}{ll} Risk\;Score & =\left(-0.222606222\times CALR\;eExpression\;level\;\right)\\ & \;+\left(-0.531973651\times CDK6\;expression\;level\right)\\ & \;+\left(0.106253563\times HOXA9\;expression\;level\right)\\ & \;+\left(0.994626253\times PARP1\;expression\;level\right) \end{array}$$

The risk coefficients are shown in Table 2.

**TABLE 2:** Risk model gene coefficients.

Gene	Coef	Exp (coef)	Se (coef)	Z	Pr (>|z|)
CALR	−0.222606222	0.800429979	0.157474682	−1.413600074	0.157479359
CDK6	−0.531973651	0.587444415	0.147764044	−3.600156287	0.000318026
HOXA9	0.106253563	1.112103829	0.059733266	1.778800481	0.075272478
PARP1	0.994626253	2.703713649	0.269146597	3.695481436	0.000219471

Note: Gene, Prognostic gene; Coef, Gene risk coefficient from stepwise regression; Exp (coef), Hazard ratio (HR); Se (coef), Standard error of HR; Z, Wald statistic (coef/se (coef)).

Risk scores were calculated for each patient based on the regression coefficients and expression levels of the prognostic genes, and the TCGA-LAML samples were categorized into high- and low-risk groups (median risk score of 0.1134133, high/low-risk patients = 66/66). Risk distribution and survival status maps showed that the high-risk group had higher risk scores and shorter survival durations than the low-risk group (Figure 3D). The KM curves showed that the high-risk group had a significantly lower probability of survival (Figure 3E). The AUC values of the ROC analyses of the 1-, 3-, and 5-year risk models were ≥0.6, suggesting a strong validity in predicting the survival of patients with AML (Figure 3F). The KM curves showed that the high-risk group had a higher risk score and a shorter survival duration than the low-risk group (Figure 3D).

In the independent cohort (GSE71014) split into high- and low-risk groups (52 samples each) with a median score of 3.269811, the high-risk group had more adverse outcomes than the low-risk group with an AUC of ≥0.6 (Figures 3G–I), validating the efficacy of the risk model.

### 3.4 Establishing a line graph for AML survival prediction based on independent risk factors

**FIGURE 4:**
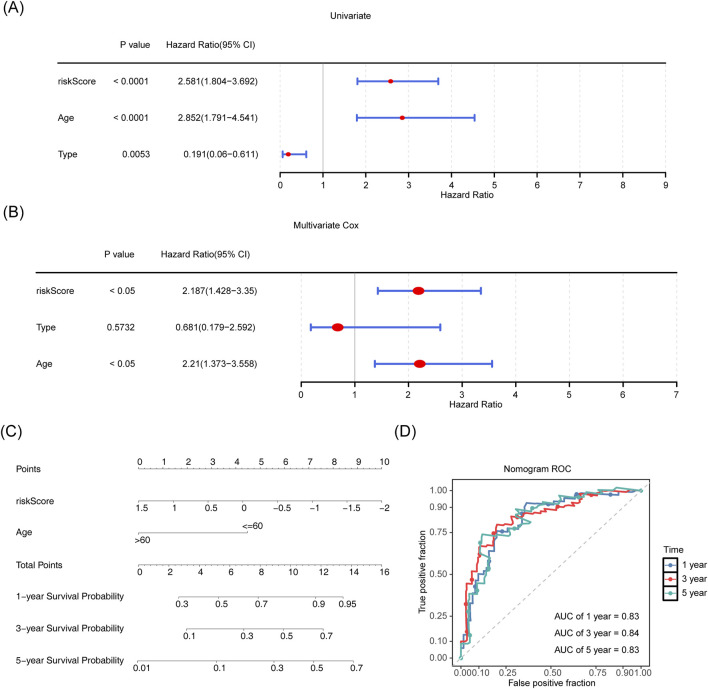
Establishing a line graph for AML survival prediction based on independent risk factors. **(A,B)** Risk score, age, and staging were included as clinical characteristics in univariate and multivariate Cox regression analyses. **(C)** Line graph for risk score and age and 1-, 3-, and 5-year survivability with AML. **(D)** ROC curves for 1-, 3- and 5-year survivability with AML.

To evaluate the prognostic model and identify independent prognostic factors for AML, a one-way Cox regression analysis (HR ≠ 1, *p* < 0.05) was performed using the risk score and age. M-staging (M3 and others) was also performed on the TCGA-LAML cohort. We found that the risk score, age, and M-stage were significantly associated with the survival of patients with AML (Figure 4A), and the PH hypothesis (*p* > 0.05) was fulfilled. The risk score and age were the independent prognostic factors in the multifactorial Cox independent prognostic analysis (Figure 4B). Based on the independent prognostic factors, a column chart was constructed with survival as the outcome event (Figure 4C). Higher scores in the column chart indicated a higher risk of death and a lower survival rate. For example, if a patient was 60 years old (points = 4.5) with a risk score of 1.5 (points = 0; total points = 4.5), the probability of surviving for 1, 3, and 5 years was approximately 50%, 20%, and 10%, respectively. ROCs were evaluated on the column line graphs, and the AUCs were all greater than 0.8, indicating good model predictions (Figure 4D).

### 3.5 Differences in tumor immune microenvironment between the high- and low-risk groups and prognostic gene expression in different cell subclusters

**FIGURE 5:**
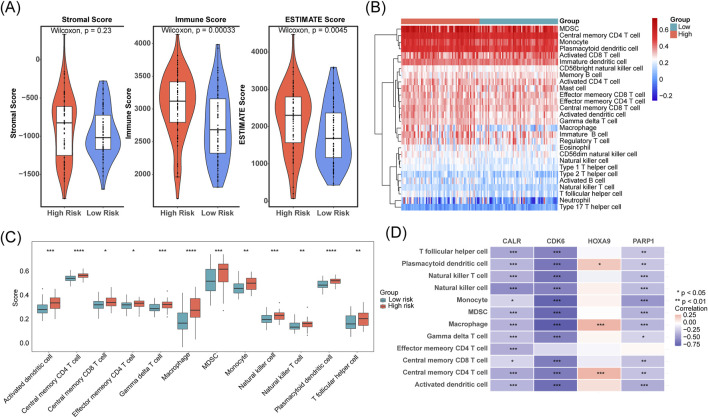
Differences in tumor immune microenvironment between the high- and low-risk groups and prognostic gene expression in different T cell subclusters. **(A)** Stromal, immune, and ESTIMATE scores were compared between low- and high-risk cohorts. **(B,C)** Immune cell infiltration levels were contrasted across low- and high-risk groups. **(D)** The correlation between prognostic genes and immune cells was assessed.

We found a significant upward trend (*p* < 0.05) in the immunization and ESTIMATE scores in the high-risk group of the TCGA-LAML cohort (Figure 5A). We compared the abundance of immune cell infiltration in the high- and low-risk groups. Twelve immune cell types showed significant differences, including activated dendritic cells, memory T-cells, and macrophages (*p* < 0.05; Figures 5B,C). Additionally, CALR, CDK6, and PARP1 were negatively correlated with most of the differential immune cells, whereas HOXA9 was positively correlated with plasmacytoid dendritic cells, macrophages, and central memory CD4^+^ T cells (*p* < 0.05; Figure 5D).

### 3.6 Functional analysis of molecular regulation of prognostic genes

**FIGURE 6:**
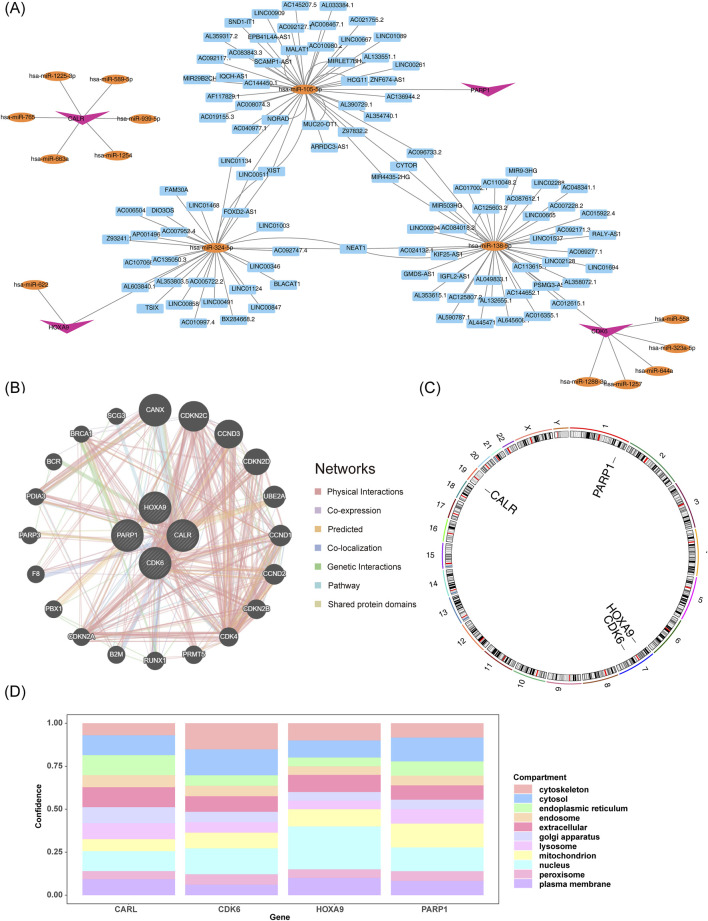
Functional analysis of molecular regulation of prognostic genes. **(A)** lncRNA-miRNA-mRNA interaction network. **(B)** Functional and co-expression analysis of prognostic genes. **(C,D)** Genomic and subcellular mapping of prognostic genes.

The “lncRNA-miRNA-mRNA” regulatory network showed that CALR, CDK6, HOXA9, and PARP1 predicted six, six, two, and one miRNAs, respectively. The miRNAs predicted 0, 40, 28, and 41 lncRNAs associated with prognostic genes, including the XIST-“hsa-miR-324-5p”-HOXA9 and XIST-“hsa-miR-105-5p”-PARP1 relationships (Figure 6A). The biological functions and co-expression networks of the prognostic genes were analyzed using the GeneMANIA database, with CANX, CDKN2C, and CCND3 having the strongest association with prognostic genes (Figure 6B). Chromosomal localization revealed that CALR, CDK6, HOXA9, and PARP1 were located on chromosomes nineteen, one, seven, and seven, respectively (Figure 6C). Subcellular localization of proteins revealed that the four prognostic genes were mainly expressed in the cytoplasm and nucleus (Figure 6D).

### 3.7 IC50 difference analysis and targeted drug prediction based on AML prognostic modeling

**FIGURE 7:**
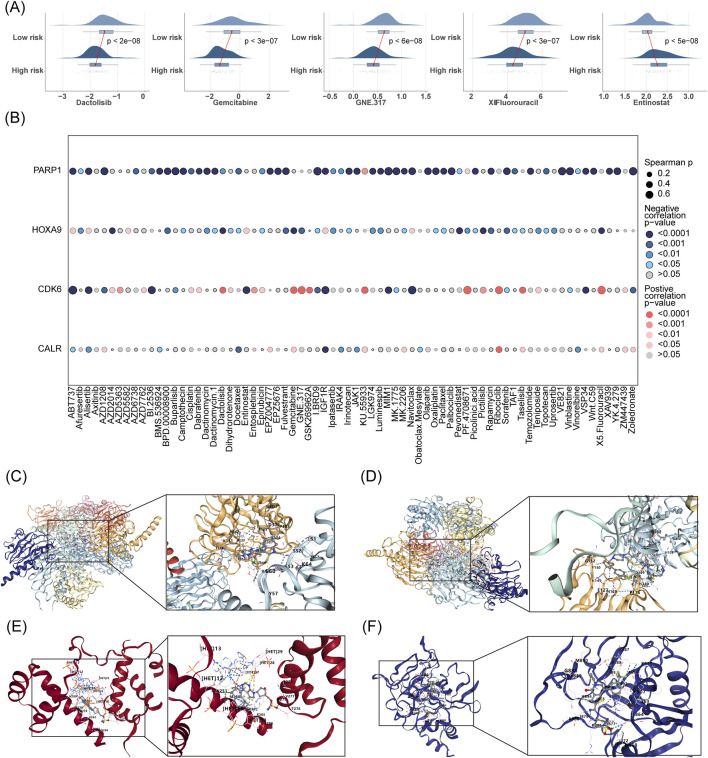
IC50 difference analysis and targeted drug prediction based on AML prognostic modeling. **(A)** Comparative IC50 analysis predicts differential drug sensitivity between low- and high-risk groups. **(B)** Assesses the correlation between prognostic gene expression and drug IC50 values. **(C–F)** Employs CB-Dock2 for molecular docking of prognostic genes to elucidate potential drug interactions.

IC50 difference analyses assess drug sensitivity. Lower IC50 values (concentration of drug required to inhibit tumor cell growth) indicate higher drug sensitivity (Garnett et al., 2012). By comparing the IC50 values of drugs in the high- and low-risk AML groups of the prediction model, we found that the high-risk group had higher sensitivity to chemotherapeutic drugs (Dactolisb, Gemcitabine, GNE.317, and 5-fluorouracil). In contrast, the low-risk group was more sensitive to entinostat (Figure 7A). Figure 7B shows the correlation between the IC50 of drugs and prognostic genes. PARP1 was negatively correlated with the IC50 of most chemotherapeutic drugs.

The prognostic genes CALR, CDK6, HOXA9, and PARP1 were identified as four targets for CB-Dock2 molecular docking, and the prognostic genes had good binding with chemotherapeutic drugs. Specifically, CALR had a binding energy of −9.9 kcal/mol with IGF1R, with residues E60 and K151 forming hydrogen bonds with IGF1R (Figure 7C). The binding energy of CDK6 to ABT737 was −10.1 kcal/mol, with residues E127, G152, and P114 forming hydrogen bonds with ABT737 (Figure 7D). The binding energy of HOXA9 to pevonedistat was −8.9 kcal/mol, with residues T276, V277, and K265 forming hydrogen bonds with pevonedistat (Figure 7E). PARP1 binds to IBRD9 with a binding energy of −10.6 kcal/mol, with residues M890, G888, N868, and S864 forming hydrogen bonds with I. BRD9 (Figure 7F, Table 3).

**TABLE 3:** Binding energy.

Gene	Drug	Binding energy
CDK6	ABT737	−10.1
PARP1	I.BRD9	−10.6
CALR	IGF1R	−9.9
HOXA9	Pevonedistat	−8.9

Note: Gene, Prognostic gene; Drug, Targeted drug for prognostic gene; Binding energy, Binding free energy between prognostic genes and chemotherapeutic drugs.

### 3.8 Experimental validation of CALR, CDK6, HOXA9, and PARP1

**FIGURE 8:**
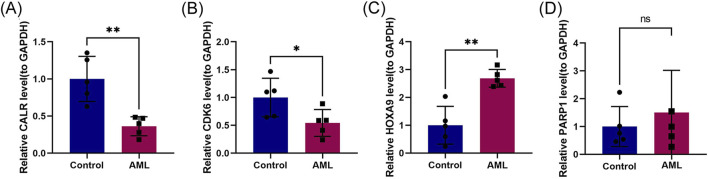
Experimental validation of CALR, CDK6, HOXA9, and PARP1. **(A–D)** RT-qPCR validation of prognostic genes CALR, CDK6, HOXA9, and PARP1 in control and refractory AML cohorts.

We validated the mRNA expression of prognostic genes by extracting RNA from fresh bone marrow from healthy individuals and patients with AML. Compared to the control group, the AML group had significantly reduced CALR and CDK6 expression and increased HOXA9 and PARP1 expression (Figure 8), consistent with the predictions of our risk model.

Insert up to 5 heading levels into your manuscript as can be seen in “Styles” tab of this template. These formatting styles are meant as a guide, as long as the heading levels are clear, Frontiers style will be applied during typesetting.

## 4 Discussion

Prior research correlates AML risk with T-cell senescence, characterized by chronic immune activation and dysfunction (Bailur et al., 2020; Chen et al., 2024; Miao et al., 2024; Song et al., 2024). In this study, we screened candidate genes using single-cell and bulk RNA sequencing data. We constructed a prognostic model using four genes (CALR, CDK6, HOXA9, and PARP1) and a line graph for predicting survival by single-factor and multifactor Cox stepwise regression using TCGA-LAML and RNA sequencing data. We also elucidated the relationship between the tumor immune microenvironment and prognostic genes, revealing that cellular senescence was significantly associated with AML. This study aimed to provide new insights for the early diagnosis, personalized treatment, and prognostication of AML.

Thirty-one candidate genes were first identified using independent external data from GEO, TCGA, and CellAge databases. The signaling pathways significantly enriched with the candidate genes were the nucleotide-binding oligomeric structural domain (NOD)-like receptor and IL-17 signaling pathways. Studies have shown that the NOD-like receptor protein 3 (NLRP3) pathway is overexpressed and highly activated in AML cells (Chen et al., 2024; Zhong et al., 2021). IL-17 stimulates the development of granulocytes and myeloid hematopoietic cells (Wei et al., 2024), and small molecules targeting the TNF/IL-17/MAPK pathway (OUL35, KJ-Pyr-9, and CID44216842) attenuate zebrafish bone marrow proliferation (Luo et al., 2024). Univariate analysis identified six prognostic predictor genes, which multivariate Cox and stepwise regression analyses further validated. Using the above analyses, we identified the prognostic genes (CALR, CDK6, HOXA9, and PARP1). The prognostic risk model categorized patients into high-risk and low-risk groups, with high-risk patients experiencing a shorter survival period. The AUC values from the ROC analyses confirmed the accuracy of the model. The qPCR validation results were consistent with the initial predictions, emphasizing the reliability of the model predictions. IC50 difference analysis yielded significant differences in sensitivity to drugs between the high- and low-risk groups in the prognostic model. Molecular docking techniques identified targeted drugs with strong binding to the prognostic genes, including pevonedistat and azacitidine (first-line therapy for AML) (Adès et al., 2022; Murthy et al., 2024; Short et al., 2023). ABT737 neutralizes the inhibition of Bax and Bak by Bcl-2, Bcl-xl, and Bcl-w at nano-micro molar concentrations, thereby inducing apoptosis (Yalniz and Wierda, 2019).

In this study, CALR, CDK6, HOXA9, and PARP1 expression in pDCs, Mφs, and central memory CD4^+^ T cells significantly differed between low- and high-risk groups. CALR induces phagocytosis by sending an “eat-me signal,” and its downregulation contributes to immune evasion in AML (Bhave et al., 2023; Liu et al., 2021; Weinhäuser et al., 2023; Zhou et al., 2024). CDK4/6 inhibitors (palbociclib) slow AML progression by reducing DNA damage and telomere shortening in T cells by inhibiting CDK4 and CDK6 kinase activities (Nebenfuehr et al., 2020; Schmoellerl et al., 2020). CDK6 knockdown stalls DN T cell differentiation at the DN3/DN4 stage, resulting in insufficient thymocytes and structural atrophy (Yabas and Hoyne, 2023). HOXA9 is a transcription factor that can be used as a prognostic indicator for AML because its dysregulation is associated with AML progression (Deshpande and Zhu, 2023; Lai et al., 2020; Salik et al., 2020; Xiao et al., 2023), specifically by recruiting EZH2 to inhibit INK4A/B (cell cycle inhibitor) expression and subsequently affecting T-cell senescence (Collins and Hess, 2016a; b). PARP1 is involved in DNA repair and gene expression, and inhibiting PARP1 has anti-tumor effects in AML (Csizmar et al., 2021; Kontandreopoulou et al., 2021; Kumar et al., 2022; Paczulla et al., 2019; Wu et al., 2023).

## 5 Conclusion

This bioinformatics-driven study identified prognostic genes linked to T-cell senescence in AML and confirmed the findings using RT-qPCR. Using public single-cell and bulk RNA-seq data, we identified 31 candidate genes associated with inflammation and hematopoiesis. A four-gene prognostic model (CALR, CDK6, HOXA9, and PARP1) was constructed using Cox and stepwise regression to forecast AML survival. Molecular docking highlighted ABT737, IBRD9, IGF1R, and pevonedistat as potential therapeutic agents with high affinity for these genes.

## Data Availability

The original contributions presented in the study are included in the article/Supplementary Material, further inquiries can be directed to the corresponding author.

## Supplementary material

SUPPLEMENTARY FIGURE S1 Quality control and dimensionality reduction clustering analysis of the single-cell sequencing dataset GSE116256.

SUPPLEMENTARY FIGURE S2 Gene difference analysis and functional enrichment of monocyte clusters (7 and 13) in the single-cell sequencing dataset GSE116256.

SUPPLEMENTARY FIGURE S3 PH hypothesis testing for six candidate genes.

SUPPLEMENTARY TABLE S1 List of CSRGs.

SUPPLEMENTARY TABLE S2 The marker gene of the different cell types.

SUPPLEMENTARY TABLE S3 Information on the control group and AML patients used for bone marrow sample collection at Suzhou Yongding Hospital.

SUPPLEMENTARY TABLE S4 List of 517 differentially expressed genes between the control group and the AML group.

SUPPLEMENTARY TABLE S5 31 cross-candidate genes, the candidate genes.
