# Supplementary figures and images for: Integrated single-cell and bulk RNA dequencing to identify and validate prognostic genes related to T Cell senescence in acute myeloid leukemia
Source: Front Bioinform. 2025 Jun 25;5:1606284. doi: 10.3389/fbinf.2025.1606284 (PMC12238043; doi:10.3389/fbinf.2025.1606284)

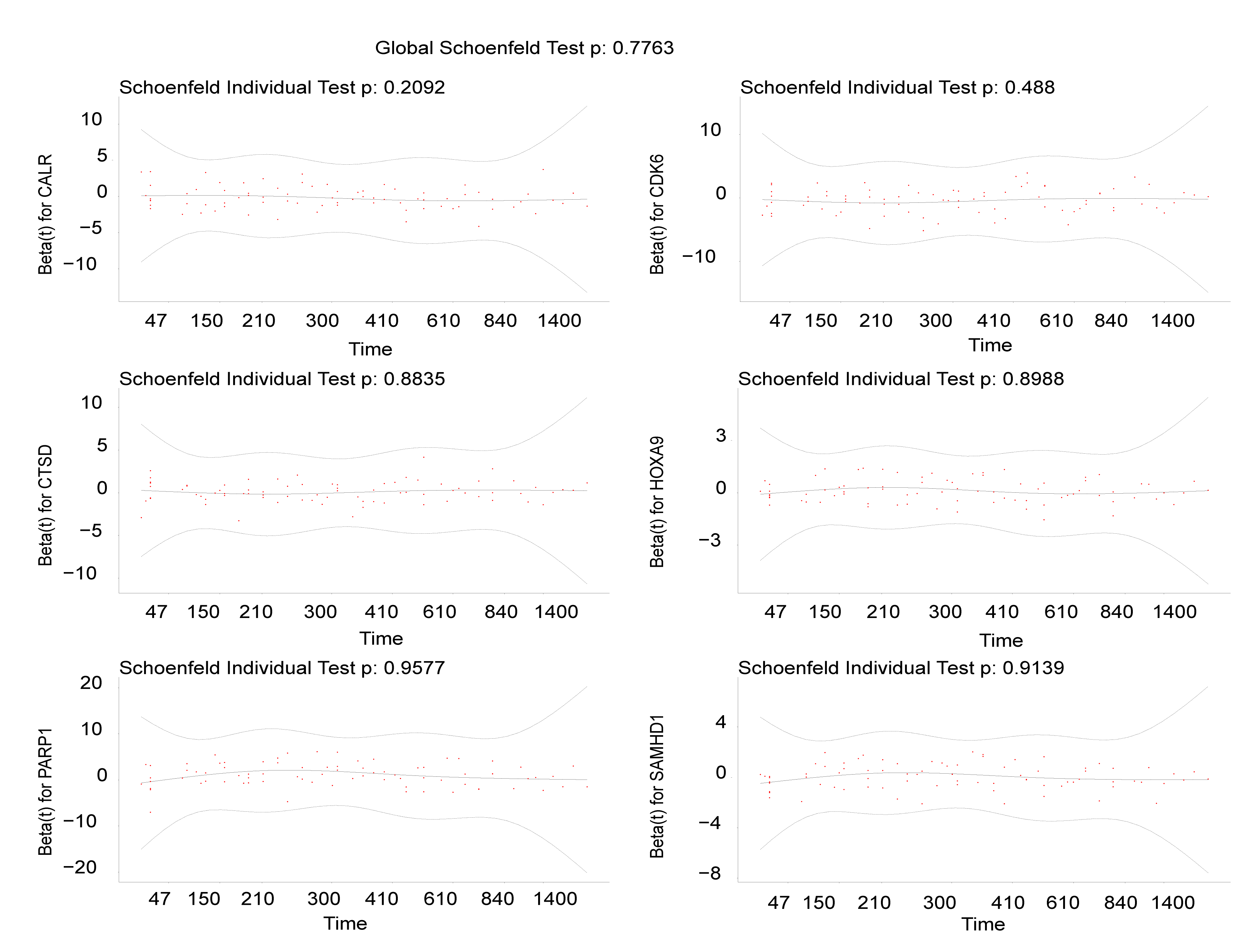

Supplement: Supplementary file 4 [file Image3.tif]

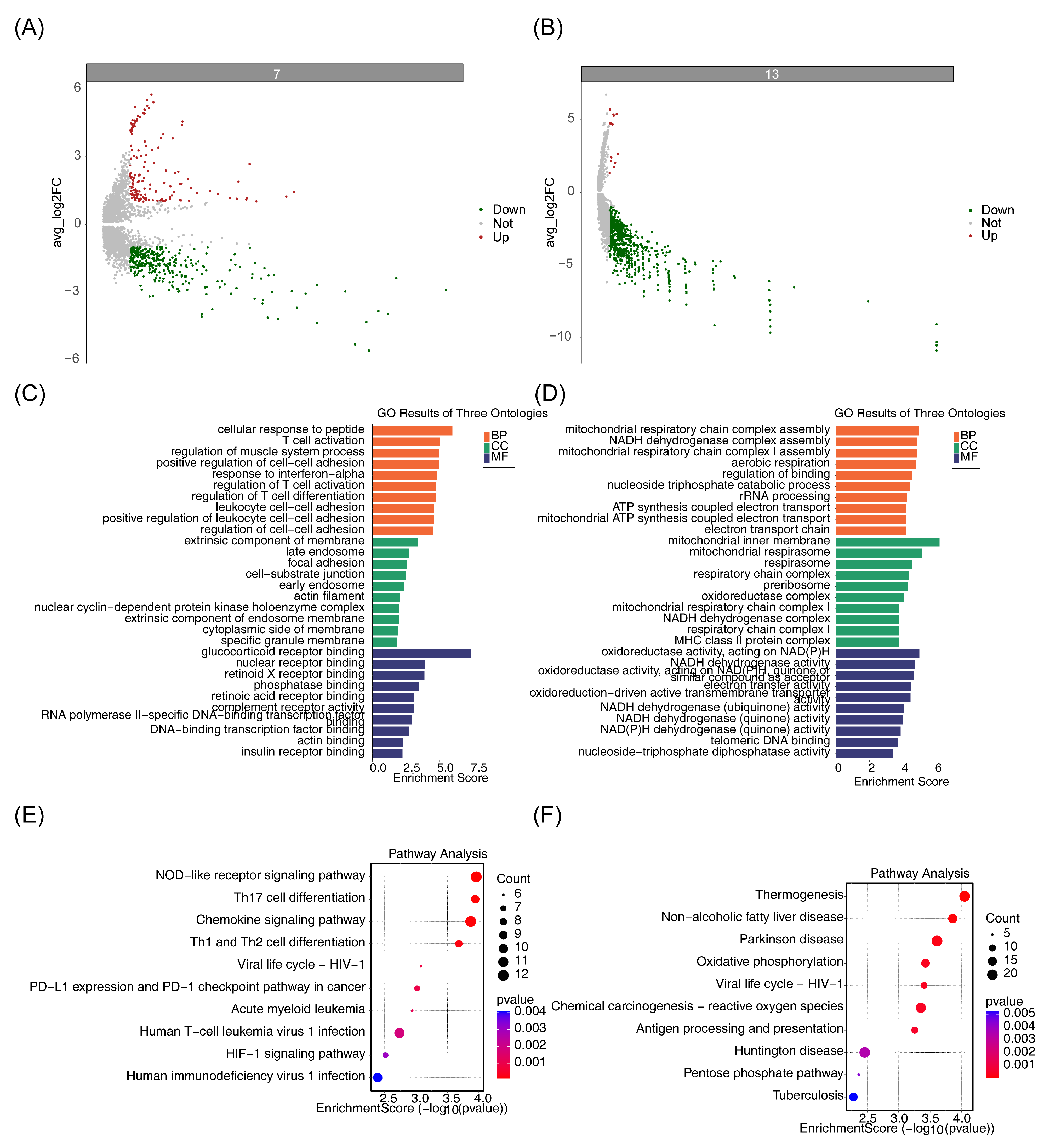

Supplement: Supplementary file 5 [file Image2.tif]

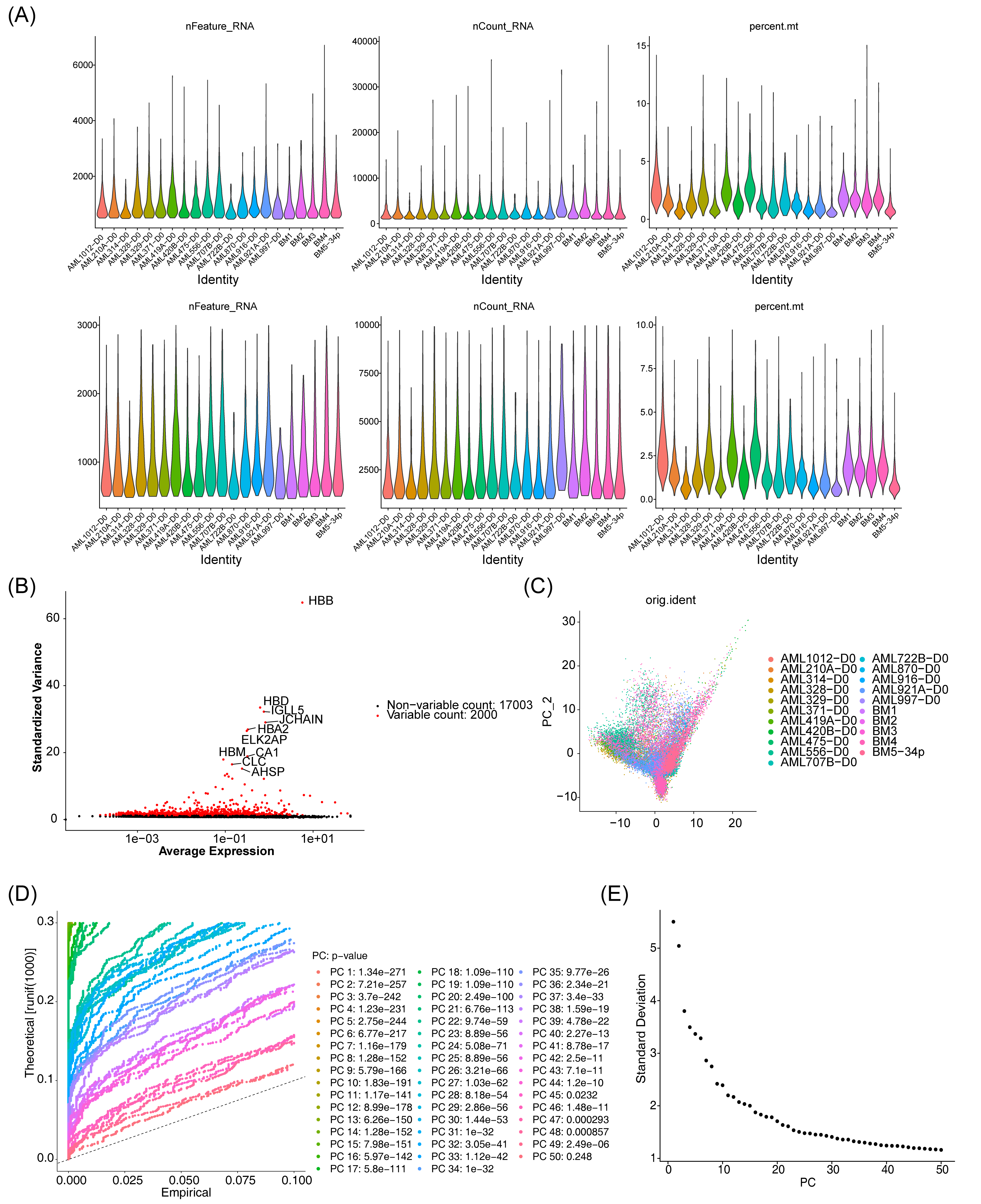

Supplement: Supplementary file 6 [file Image1.tif]
